# PRISMA extension for moxibustion 2020: recommendations, explanation, and elaboration

**DOI:** 10.1186/s13643-020-01502-7

**Published:** 2020-10-25

**Authors:** Xuan Zhang, Ran Tan, Wai Ching Lam, Chung Wah Cheng, Liang Yao, Xiao-Qin Wang, Si-Yao Li, Qi-Ying Aixinjueluo, Ke-Hu Yang, Hong-Cai Shang, Tai-Xiang Wu, Ai-Ping Lyu, Zhao-Xiang Bian

**Affiliations:** 1 Chinese Clinical Trial Registry (Hong Kong), Hong Kong Chinese Medicine Clinical Study Centre, School of Chinese Medicine, Hong Kong Baptist University, Hong Kong, China; 2 Chinese EQUATOR Centre, Hong Kong Baptist University, Jockey Club School of Chinese Medicine Building, 7 Baptist University Road, Kowloon Tong, Kowloon, Hong Kong, SAR China; 3 Evidence-Based Medicine Center, School of Basic Medical Sciences, Lanzhou University, Lanzhou, China; 4 Key Laboratory of Chinese Internal Medicine of Ministry of Education and Beijing, Dongzhimen Hospital, Beijing University of Chinese Medicine, Beijing, China; 5 Chinese Cochrane Centre, West China Hospital, China Trial Registration Center, Sichuan University, Chengdu, Sichuan China

**Keywords:** Chinese medicine, Extension, Moxibustion intervention, PRISMA, Systematic review

## Abstract

**Background:**

Moxibustion is a common intervention of Chinese medicine (CM). Systematic reviews (SRs) on moxibustion are increasing. Although the Preferred Reporting Items for Systematic Reviews and Meta-Analyses (PRISMA) statement provides guidelines for SRs, the quality of moxibustion-related SRs is still not satisfactory. In particular, descriptions of the interventions and the rationale for using moxibustion are insufficient. To address these inadequacies, the working group developed this PRISMA extension for reporting SRs of moxibustion (PRISMA-M 2020).

**Methods:**

A group of CM clinical professionals, methodologists of SRs, reporting guideline developers, and journal editors developed this PRISMA-M 2020 through a comprehensive process that includes registration, literature review, consensus meetings, Delphi exercises for soliciting comments, and revision, resulting in this final draft.

**Results:**

Seven of the 27 PRISMA checklist items, namely title (1), rationale (3), eligibility criteria (6), data item (11), additional analyses (16), study characteristics (18), and additional analysis (23), were extended, with specific reference to the application of moxibustion. Illustrative examples and explanations for each item are provided.

**Conclusion:**

The PRISMA-M 2020 will help improve the reporting quality of SRs with moxibustion.

**Systematic review registration:**

We have registered it on the EQUATOR (Enhancing the QUAlity and Transparency Of health Research) network, particularly under the item of PRISMA-TCM: http://www.equator-network.org/library/reporting-guidelines-under-development/reporting-guidelines-under-development-for-systematic-reviews/#65.

**Supplementary Information:**

The online version contains supplementary material available at 10.1186/s13643-020-01502-7.

## Background

Moxibustion is a traditional therapeutic technique in which hot or burning moxa is applied on acupoints or meridians [1]. The earliest record of moxibustion is on the oracle bones, demonstrating that moxibustion had occurred in the Yin dynasty (1600–1046 BC) [2]. According to relics excavated from the Mawangdui tomb and Hantanpo tomb, moxibustion was used to treat many diseases and had a general application in the Qin and Han dynasties (221 BC–220 AD) in China [3]. In the practice of Chinese medicine (CM), moxibustion is a valuable and unique type of CM intervention. With the functions of warming the meridians, promoting a smooth flow of Qi and blood, relieving the obstruction of collateral vessels, and regulating the zang-fu organs, moxibustion has been used to treat and prevent diseases, as well as maintain health and well-being for more than 2500 years [4–6]. Nowadays, the practice of moxibustion has been, and continues to be, widely used in Asia (mainly in China, Japan, Korea), and its use is increasing in North America and Europe [7, 8].

With more and more randomized controlled trials (RCTs) and systematic reviews (SRs) of moxibustion published every year, the importance of improving the reporting quality of moxibustion studies has been highlighted by both researchers and users of moxibustion evidence [9–11]. In 2013, our research group developed the STandards for Reporting Interventions in Clinical Trials Of Moxibustion (STRICTOM) [12], as an extension of the Consolidated Standards of Reporting Trials (CONSORT) [13]. Some scholars have noted that the reporting quality of clinical trials of moxibustion improved after the implementation of STRICTOM [14, 15]. However, no specific reporting guidelines for moxibustion SRs have been developed, although the PRISMA statement has been published for many years [16], and several extensions, such as PRISMA for Abstracts [17], Protocols [18], Harms [19], and Complex Interventions [20], have been developed.

Recently, the quality of reporting of SRs with various CM interventions has raised concern, and relevant guidelines have been developed, such as PRISMA for acupuncture [21] and PRISMA for Chinese herbal medicines [22]. These extensions highlight how intervention details and the CM-based rationale of why the interventions were selected, such as “treatment based on pattern differentiation,” should be reported. Although PRISMA provides the general reporting requirements of “Rationale” and “Intervention” items for all types of SRs, relevant item extensions are needed for specific types of CM SRs because each CM intervention has unique characteristics that must be considered in order to provide high-quality evidence for clinical practice.

Given this situation, we suspected that the practices of moxibustion were not being adequately reported, despite PRISMA guidelines. Aiming to determine if this were true, we conducted a preliminary literature review of 97 moxibustion SRs from 2011 to 2019. In the results, 84.5% (82/97) SRs studied CM-based moxibustion interventions and published the results in Chinese journals. For the reporting of intervention technique, 69.1% (67/97) SRs did not provide the specific type of moxibustion; 39.2% (38/97) lacked details regarding the materials, procedure, and technique used for moxibustion; 67.0% (65/97) did not fully report the selection of acupoints for moxibustion; and 28.9% (28/97) did not provide the number or duration of treatment sessions [23]. Given such inadequate reporting, readers cannot assess the data synthesis nor the conclusions of these SRs. Such a situation undermines the value, use, and development of moxibustion therapy in modern medical practice. To address this inadequacy, our working group therefore developed a reporting extension for SRs with moxibustion based on the PRISMA checklist.

## Methods

The PRISMA-M 2020 is based on the PRISMA statement. The methodology framework recommended by the EQUATOR Network was used in developing it [24], in the following seven steps:

### Registration

This PRISMA-M was included in the registered item of “PRISMA-TCM” on the EQUATOR Network, as moxibustion is a common and important type of traditional Chinese medicine [25].

### Literature review

Authors XZ, S-YL, Q-YA, and RT conducted a literature review to assess the reporting quality of moxibustion SRs and continued to update the included articles until 31 December 2019 (Additional file 1: S1). The results of this review guided the preliminary drafting of the items ultimately included in the extension [23].

### Items extraction

Authors XZ, ZX-B, and TX-W extracted the initial PRISMA items that needed extension or modification according to the characteristics of moxibustion SRs. Further, the working group members (see author list) reviewed all potential items and formulated a reporting checklist on 15 June 2017.

### Consensus meeting

A total of eleven professionals, including five senior TCM practitioners, three evidence-based medicine and clinical trial methodology experts, two reporting guideline developers, and one epidemiologist, were invited to attend a face-to-face meeting in Lanzhou, China, on 19 July 2017. During the meeting, the aim and scope of the guideline with a drafted checklist were presented to the participants, followed by a discussion and revision of each proposed item. Finally, a checklist questionnaire was formed for a Delphi survey for further solicitation of comments.

### Delphi exercise

Three rounds of an e-mail-based Delphi survey were conducted from November 2017 to April 2018. Thirty-five experts with rich experience in moxibustion practice, performing SRs, and developing reporting guidelines were invited. The invitation letters, which included an introduction to the study, workflow of Delphi surveys, and relevant questionnaire, were sent out; positive responses confirmed their participation. Each of the 35 individuals completed at least two rounds of the Delphi survey. The questionnaires of each round were sent to the participants from a specific email address, which was managed by one member (RT) of the working group. Anonymity and confidentiality of responses were ensured.

During the Delphi process, participants were asked to rate each item on a scale of 1 (not important) to 5 (very important), to suggest new items, and to provide comments; any items that did not reach consensus (e.g., score < 75%) and any new items were circulated in subsequent rounds. Following each round, the score for each item was calculated with the formula 100% × (1 × *N*_5_ + 0.75 × *N*_4_ + 0.5 × *N*_3_ + 0.25 × *N*_2_)/(*N*_5_ + *N*_4_ + *N*_3_ + *N*_2_ + *N*_1_), where *N*_*i*_ represents the number of respondents who chose specific *i* in the scale of “1 to 5.” Items with a score greater than or equal to 75%, namely reach a consensus, were included [26]. This calculation formula was referred to the RIGHT (A Reporting Tool for Practice Guidelines in Health Care) statement, where both the consensus level and the weight of responses were considered [27]. The analyses of the included items were managed by authors RT, XZ, and ZX-B.

### Explanation and elaboration preparation

Explanation and elaboration (E&E) documents were prepared for each included item. Examples of good reporting were identified and edited for inclusion (e.g., citations or web addresses were removed; abbreviations were spelled out). Explanation documents were also developed to provide the rationale and describe the characteristics of moxibustion SRs.

### Revision and finalization

The manuscript of this guideline was drafted by authors ZX-B and XZ. The working group members reviewed and provided revisions. The draft was also presented by team leaders in academic workshops and conferences to solicit broad comments for improvement [28]. The manuscript, including recommendations and E&E, was finalized in May 2020.

## Results

### Highlights of PRISMA-M 2020

PRISMA-M 2020 expands sections of PRISMA to ensure that the unique characteristics of moxibustion are adequately reported in SRs, so as to ensure that the data and results are accurate and complete, and that the study is reproducible. Also, the key concept of CM pattern (Table 1) is highlighted in the practice of moxibustion. Specifically, PRISMA-M 2020 elaborates on 7 of 27 PRISMA original items, namely title (1), rationale (3), eligibility criteria (6), data items (11), additional analyses (16), study characteristics (18), and additional analysis (23). The items from the STRICTOM [12] and Template for Intervention Description and Replication (TIDieR) [29] are also included, especially for the details of moxibustion intervention. The PRISMA-M 2020 checklist is presented in Table 2; extensions for moxibustion are italicized. Explanations of corresponding items are given below, and available reporting examples are provided in Additional file 1: S_2_. There is no modification of the PRISMA flow diagram.

**Table 1:** Fundamental principles and methods of CM theory: pattern identification [30, 31]

The Chinese medicine (CM) theoretical system evolved over centuries. Its fundamental principle is that the determination of CM interventions must be based on pattern differentiation (also called syndrome differentiation, or “Bian-Zheng-Lun-Zhi” in Chinese), a primary CM method of understanding and treating diseases. According to CM theory, a pattern (also termed a syndrome or “Zheng” in Chinese) is a pathological cluster or summary of signs and symptoms at a particular stage of a disease. The pattern may include the cause(s), pathological features, properties, and the relationship between any pathogens involved and the body’s resistance. The patterns are named according to a cluster of associated signs and symptoms described in terms of yin, yang, exterior, interior, cold, heat, deficiency, and excess. In general, a pattern is composed of “location of disease” and “feature of disease.” A “pattern” (Zheng) is obtained through analyzing the “symptoms,” while the “disease,” especially in Western terms, comprises the whole morbid process and may include several different patterns. Specifically, pattern differentiation refers to the analysis and summarization of the clinical symptoms obtained through the four diagnostic methods of CM (inspection, auscultation and smell, inquiry, and pulse taking and palpation), after which CM practitioners can accordingly determine the specific treatment. In practice, one disease may include several different CM patterns, and conversely, different diseases may exhibit the same CM pattern in the course of their development. Thus, the application of pattern differentiation may “treat the same diseases with different methods,” or it may “treat different diseases with the same therapeutic method.” Accurate CM pattern differentiation is critical. It provides a diagnostic label, it guides the choice of CM interventions, such as moxibustion, and it gives access to the historical record of the treatments other doctors over centuries have used. In clinical practice, pattern diagnosis can help the practitioner determine a treatment principle and methods of moxibustion therapy, including the selection of acupoints, types, materials, and techniques. For example, the treatment principles of moxibustion used for excessive syndrome and deficiency syndrome are very different. For SRs of moxibustion, if the primary studies included pattern differentiation, the concept of the studied pattern should be carried out throughout the entire process with regard to the rationale of the review design, inclusion and exclusion criteria, selection of moxibustion intervention(s), outcomes, data interpretation and additional analyses, etc.

**Table 2:** Checklist of items for reporting systematic reviews of moxibustion*

Section/topic	Item number	PRISMA original item	Extension for moxibustion	Reported on page number
Title				
Title	1	Identify the report as a systematic review, meta-analysis, or both	*1a. Statement of the specific type of moxibustion treatment, such as direct moxibustion or heat-sensitive moxibustion.* *1b. Statement of whether the review targets the (1) Western medicine–defined disease(s), (2) Western medicine–defined disease(s) with specific CM pattern(s), or (3) CM pattern(s), if applicable.*	
Abstract				
Structured summary	2	Provide a structured summary including, as applicable: background; objectives; data sources; study eligibility criteria, participants, and interventions; study appraisal and synthesis methods; results; limitations; conclusions and implications of key findings; systematic review registration number		
Introduction				
Rationale	3	Describe the rationale for the review in the context of what is already known	*Describe the rationale for what is already known about moxibustion utilized for the target disease and/or CM pattern (if any). If applicable, relevant theory of CM should be included.*	
Objectives	4	Provide an explicit statement of questions being addressed with reference to participants, interventions, comparisons, outcomes, and study design (PICOS)		
Methods				
Protocol and registration	5	Indicate if a review protocol exists, if and where it can be accessed (e.g., web address), and, if available, provide registration information including registration number		
Eligibility criteria	6	Specify study characteristics (e.g., PICOS, length of follow-up) and report characteristics (e.g., years considered, language, publication status) used as criteria for eligibility, giving rationale	*6a. Describe the diagnostic criteria of the target condition in Western medicine and/or CM pattern (if any). All criteria utilized should be universally recognized, or reference(s) where detailed explanation can be found should be given.* *6b. Specify the types of moxibustion to be included, such as moxa burner moxibustion, natural moxibustion, or heat-sensitive moxibustion.* *6c. State whether CM-related outcome(s) were included, such as the change of degree and scope of symptoms and signs related to CM pattern, or validated pattern survey, if applicable.*	
Information sources	7	Describe all information sources (e.g., databases with dates of coverage, contact with study authors to identify additional studies) in the search and date last searched		
Search	8	Present full electronic search strategy for at least one database, including any limits used, such that it could be repeated		
Study selection	9	State the process for selecting studies (i.e., screening, eligibility, included in the systematic review, and, if applicable, included in the meta-analysis)		
Data collection process	10	Describe the method of data extraction from reports (e.g., piloted forms, independently, in duplicate) and any processes for obtaining and confirming data from investigators		
Data items	11	List and define all variables for which data were sought (e.g., PICOS, funding sources) and any assumptions and simplifications made	*11a. List and define the data of CM pattern(s) in detail, considering the inclusion and exclusion criteria, if applicable.* *11b. List and define the data of moxibustion interventions and controls (e.g., sham moxibustion), give details referring to STRICTOM and TIDieR.* *11c. List and define the data of CM pattern outcome(s), considering the methods and timepoints, if applicable.*	
Risk of bias in individual studies	12	Describe methods used for assessing risk of bias of individual studies (including specification of whether this was done at the study or outcome level) and how this information is to be used in any data synthesis		
Summary measures	13	State the principal summary measures (e.g., risk ratio, difference in means)		
Synthesis of results	14	Describe the methods of handling data and combining results of studies, if done, including measures of consistency (e.g., *I*^2^) for each meta-analysis		
Risk of bias across studies	15	Specify any assessment of risk of bias that may affect the cumulative evidence (e.g., publication bias, selective reporting within studies)		
Additional analyses	16	Describe methods of additional analyses (e.g., sensitivity or subgroup analyses, meta-regression), if done, indicating which were pre-specified	*Describe methods of subgroup analyses in terms of different types of included moxibustion interventions and/or included CM pattern participants (if applicable), if done, indicating which were pre-specified.*	
Results				
Study selection	17	Give numbers of studies screened, assessed for eligibility, and included in the review, with reasons for exclusions at each stage, ideally with a flow diagram		
Study characteristics	18	For each study, present characteristics for which data were extracted (e.g., study size, PICOS, follow-up period) and provide the citations	*18a. Present characteristics for the data of participants, which include CM pattern(s), considering (1) diagnostic criteria; (2) baseline data, if applicable.* *18b. Present characteristics for the data of moxibustion intervention(s) and controls (e.g., sham moxibustion) for each study referring to STRICTOM and TIDieR.* *18c. Present characteristics for the data of outcomes which include CM pattern(s), considering (1) name and measuring methods; (2) measuring timepoints and length of follow-up, if applicable.*	
Risk of bias within studies	19	Present data on the risk of bias of each study and, if available, any outcome-level assessment (see item 12)		
Results of individual studies	20	For all outcomes considered (benefits or harms), present, for each study: (a) simple summary data for each intervention group and (b) effect estimates and confidence intervals, ideally with a forest plot		
Synthesis of results	21	Present results of each meta-analysis done, including confidence intervals and measures of consistency		
Risk of bias across studies	22	Present results of any assessment of the risk of bias across studies (see item 15)		
Additional analysis	23	Give results of additional analyses, if done (e.g., sensitivity or subgroup analyses, meta-regression [see item 16])	*Give results of subgroup analyses based on the different types of moxibustion interventions and participants with CM patterns (if any), if done.*	
Discussion				
Summary of evidence	24	Summarize the main findings including the strength of evidence for each main outcome; consider their relevance to key groups (e.g., health care providers, users, and policymakers)		
Limitations	25	Discuss limitations at study and outcome level (e.g., risk of bias) and at review level (e.g., incomplete retrieval of identified research, reporting bias)		
Conclusions	26	Provide a general interpretation of the results in the context of other evidence, and implications for future research		
Funding				
Funding	27	Describe sources of funding for the systematic review and other support (e.g., supply of data); role of funders for the systematic review		

*The original PRISMA items are provided; elaborations for moxibustion interventions are in italicized text. We strongly recommend reading this checklist in conjunction with the PRISMA 2009 explanation and elaboration for important clarifications of the 27 items of PRISMA [32]

### Aim and scope of PRISMA-M 2020

The aim of PRISMA-M 2020 is to optimize the reporting of SRs focusing on moxibustion interventions for specific conditions and/or patterns (if any). We consider most of the PRISMA items relevant in reporting SRs assessing the benefits and harms of specific moxibustion treatments. Further, we emphasized the concept of CM pattern (if applicable) since the moxibustion prescription in CM clinical practice can be determined on pattern identification. However, we recognize that authors who address the research question without pattern contents and/or no pattern-related indicators in the primary studies may not need to focus on pattern-related extension items for the SRs. To maximize the clarity of this checklist, examples and explanations are provided for each item. For certain thematic concepts (e.g., pattern differentiation), a comprehensive description is presented in Table 1. We believe this PRISMA-M 2020 checklist will be a valuable tool for the authors and users of SRs of moxibustion.

### Explanations of PRISMA-M 2020 items

#### Title

##### Item 1: Title

PRISMA item: Identify the report as a systematic review, meta-analysis, or both.

*Extension: 1a. Statement of the specific type of moxibustion treatment, such as direct moxibustion or heat-sensitive moxibustion. 1b. Statement of whether the review targets the (1) Western medicine–defined disease(s), or (2) Western medicine–defined disease(s) with specific CM pattern(s), or (3) CM pattern(s), if applicable.*

*Explanation*. For SRs, a self-explanatory title including the PICOS (participants, interventions, comparators, outcomes, and study design) can make vital information easily accessible to readers. If the review investigates the effect of a specific type of moxibustion on a particular condition, it should be stated in the title. There are different types of moxibustion regarding the materials used and the details of procedures, such as moxa cone moxibustion, warming needle moxibustion, natural moxibustion, and heat-sensitive moxibustion [33]. Authors are strongly encouraged to use and provide standard terminologies of moxibustion released by the World Health Organization (WHO) or International Organization for Standardization (ISO) [34, 35]. If an SR aims to target a large category of moxibustion, it is better to point this out in the title and to report, in detail, the types of moxibustion in the Abstract.

Whether the moxibustion targets a Western medicine–defined disease and/or specific pattern(s) should be clarified in the title. Readers can then easily understand which conditions are studied in the SR, and the results will be more easily applied in clinical practice. In terms of reporting the pattern names, authors are encouraged to provide the specific name if one type of pattern that will be targeted, such as “primary dysmenorrhea with cold coagulation and blood stasis syndrome” [36] and “prostatitis (hot and humid stasis syndrome)” [37]. If an SR includes a broad category of patterns (e.g., more than two types of pattern), it is better to use a generalized term in the title [38], such as “Patterns,” “Pattern-based,” and “Pattern identification.”

#### Introduction

##### Item 3: Rationale

PRISMA item: Describe the rationale for the review in the context of what is already known.

*Extension: Describe the rationale for what is already known about moxibustion utilized for the target disease and/or CM pattern (if any). If applicable, relevant theory of CM should be included.*

*Explanation*. CM has long been thought to be a kind of personalized medicine, and its unique characteristic is pattern differentiation [30, 31]. In actual clinical practice, Pattern diagnosis can help the practitioner determine a treatment principle and methods of moxibustion therapy, including the selection of acupoints, types, materials, and techniques [39]. For example, the treatment principles of moxibustion used for excessive syndrome and deficiency syndrome are very different. Similar to CM theory, other traditional medicine systems, such as Korean medicine, also emphasize individuality during moxibustion treatment [40]. Therefore, for moxibustion SRs, it is necessary to provide relevant CM (or other traditional medicine) theories in the description of studied disease(s)/pattern(s) and intervention(s). It is also recommended to include any medical rationale as to “how the intervention might work” in Western scientific terms. Such information can help readers to realize the importance of the research question in the review.

#### Methods

##### Item 6: Eligibility criteria

PRISMA item: Specify study characteristics (e.g., PICOS, length of follow-up) and report characteristics (e.g., years considered, language, publication status) used as criteria for eligibility, giving rationale.

*Extension: 6a. Describe the diagnostic criteria of the target condition in Western medicine and/or CM pattern (if any). All criteria utilized should be universally recognized, or reference(s) where detailed explanation can be found should be given. 6b. Specify the types of moxibustion to be included, such as moxa burner moxibustion, natural moxibustion, or heat-sensitive moxibustion. 6c. State whether CM-related outcome(s) were included, such as the change of degree and scope of symptoms and signs related to CM pattern, or validated pattern survey, if applicable.*

*Explanation*. Diagnostic criteria of disease(s)/pattern(s) are important to clarify the scope of target populations in the review. How the disease and/or pattern (if any) was diagnosed and what criteria were used for identifying participants should be comprehensively described. In addition, because moxibustion can be used both to treat disease and to improve health, the condition diagnosed should be clearly identified as either a disease or simply a less-than-optimum state of health. Generally, authors should choose nationally or internationally recognized diagnostic criteria and then include the eligible primary studies. However, there may be different diagnostic criteria for one pattern as established by different original trials [41]. Thus, specific eligibility criteria for the SR should be clearly pre-designed and comprehensively described if the pattern will be involved in participant selection.

The effects of different types of moxibustion can differ. Because of this, authors should pre-define the inclusion criteria for the studied intervention. If a specific type of moxibustion will be performed, it is suggested that authors should report eligibility criteria with standard terms and definitions (if necessary), materials, procedures, and techniques. If a broad type of moxibustion will be included, authors should pre-list its scope in as much detail as possible. Additionally, moxibustion is sometimes combined with other therapies, such as acupuncture, herbs, or cupping. Thus, where applicable, authors of SRs should pre-define the selection scope and criteria for complex interventions using information from original trials.

For clinical trials testing the efficacy of moxibustion, the outcomes can usually be categorized into Western medicine–specific outcomes and CM-specific outcomes. Therefore, if an SR will include CM-related outcome(s) as eligibility criteria, the requirements for measurements and time points should be specified, because one pattern-outcome may have been assessed with different measurements in different studies [42].

##### Item 11: Data items

PRISMA item: List and define all variables for which data were sought (e.g., PICOS, funding sources) and any assumptions and simplifications made.

*Extension: 11a. List and define the data of CM pattern(s) in detail, considering the inclusion and exclusion criteria, if applicable. 11b. List and define the data of moxibustion interventions and controls (e.g., sham moxibustion), give details referring to STRICTOM and TIDieR. 11c. List and define the data of CM pattern outcome(s), considering the methods and timepoints, if applicable.*

*Explanation*. If the CM patterns were involved in selecting participants, information on how the pattern was diagnosed and what criteria were used for inclusion and exclusion should be reported in detail. If applicable, other information, such as numbers in different patterns groups, should be included in the data. If pattern-related outcomes are assessed, it is recommended to use symptoms and signs that can be measured objectively, such as in terms of occurrence (e.g., presence or absence of symptoms or signs), by a rating scale (e.g., score assessment), or an assessment questionnaire (e.g., validated pattern survey) [43, 44]. In this way, pattern measurements used in different trials can be more readily compared. In addition, the name(s) and procedure(s) of the method(s), supporting rationale or reference(s) (if any), and the evaluators (e.g., CM practitioners, trainers, or self-reporting of patients) should be reported.

For moxibustion, the effect is associated with several important details, such as the materials and techniques used, the acupoints and meridians selected, and the number, frequency, and duration of treatment; all of these factors can vary between diseases and/or patterns. If sham moxibustion is included, descriptions on how to assess whether “sham” was achieved should be particularly reported [45]. To ensure accurate and transparent reporting, it is recommended that authors report these details of moxibustion according to the checklists of STRICTOM and TIDieR guidelines [12, 29]. Although some detailed information may be recorded as “not reported” in the section of results (e.g., Item 18: Study characteristics), it is essential to pre-define all variables for data items in the section of methods.

##### Item 16: Additional analyses

PRISMA item: Describe methods of additional analyses (e.g., sensitivity or subgroup analyses, meta regression), if done, indicating which were pre-specified.

*Extension: Describe methods of subgroup analyses in terms of different types of included moxibustion interventions and/or included CM pattern participants (if applicable), if done, indicating which were pre-specified.*

*Explanation*. Whether subgroup analysis should be conducted generally depends on the review objective and the heterogeneity of interventions and participants. Due to the characteristics of moxibustion interventions and CM patterns (if any), we suggest that authors consider at least the following factors in subgroup analyses: (1) different types and/or amounts of moxibustion, (2) different treatment frequency and/or courses, (3) different phases of diseases and/or patterns (if any), and (4) different control groups. If one specific type of moxibustion is studied in an SR, more details of the factors (e.g., different types of materials, treatment acupoints, and durations) should be considered in heterogeneity analyses. Although the information may be inadequately reported in original studies, it is important for reviewers to inform readers whether the subgroup analyses were pre-specified and the rationale.

#### Results

##### Item 18: Study characteristics

PRISMA item: For each study, present characteristics for which data were extracted (e.g., study size, PICOS, follow-up period) and provide the citation.

*Extension: 18a. Present characteristics for the data of participants, which include CM pattern(s), considering (1) diagnostic criteria; (2) baseline data, if applicable. 18b. Present characteristics for the data of moxibustion intervention(s) and controls (e.g., sham moxibustion) for each study referring to STRICTOM and TIDieR. 18c. Present characteristics for the data of outcomes which include CM pattern(s), considering (1) name and measuring methods; (2) measuring timepoints and length of follow-up, if applicable.*

*Explanation*. Results should be presented referring to the pre-designed data items (e.g., item 11). If any data item is insufficiently reported and cannot be obtained by contacting authors of the included trials, then it should be described as “not reported” in the review. For moxibustion SRs, information about the CM pattern (if any) studied and about interventions should be completely provided in the results, as this is the basis for readers’ assessment of the validity of data synthesis in the SRs [46]. It is highly recommended that authors summarize the details of all relevant information for each included study in tables, which can be shown in the appendix or supplementary files. Such a presentation ensures that all pertinent items are reported and that missing or unclear information is also noted. If some data items outside the pre-defined items (e.g., item 11) are reported in the results, authors should explain why they were not included in those pre-defined items and should give all the relevant characteristics, especially with regard to interventions and patterns, if any.

##### Item 23: Additional analysis

PRISMA item: Give results of additional analyses, if done (e.g., sensitivity or subgroup analyses, meta-regression [see item 16]).

*Extension: Give results of subgroup analyses based on the different types of moxibustion*

*interventions and participants with CM patterns (if any), if done.*

*Explanation*. If subgroup analyses are done, authors should give the results, even those without statistical significance, and they should state whether the items analyzed were pre-specified (see Items 16). Subgroup analyses are valuable because the different types of moxibustion and patterns (if any) are primary sources of heterogeneity across included studies [47]; however, it is essential to have sufficient relevant data for subgroup analysis. If any predefined subgroup analysis cannot be performed, authors should provide reasons (e.g., limited or insufficient data) to avoid selective outcome reporting bias.

## Discussion

SRs of moxibustion are essential for evaluating the efficacy and safety of this unique from of medical intervention; they are expected to be accurate and reliable. With the aim of improving their reporting quality, this PRISMA for moxibustion extension was specifically developed for SRs studying CM-based moxibustion, taking into account the pattern concept on the basis of clinical practice. Other moxibustion types of traditional medicine (e.g., Korean moxibustion), or non-pharmacological interventions that include moxibustion, could also use this as a basis for reporting. Generally, we expect that the main users of this guideline will be authors of SRs on moxibustion, journal editors, peer reviewers, methodologists, and clinical moxibustion practitioners.

As with other extensions, the PRISMA-M 2020 checklist should be used together with the original PRISMA checklist. To facilitate this use, Table 2 shows a combined checklist including both the extended moxibustion items and the original PRISMA items. To maximize the clarity of this checklist, explanations and elaborations are provided for each item, including rationale for extensions, moxibustion characteristics, and the necessity for reporting. In addition, each item is presented with two or more examples of good reporting, covering a wide range of moxibustion in complementary and alternative medicine (CAM), although the majority are CM-based studies. The examples were extracted from published SRs in both Chinese and international journals. It is recommended that users read all examples listed for one specific item to get a comprehensive understanding.

For better dissemination of PRISMA-M 2020, we will take the following specific steps: Firstly, we will continue to share it with clinical practitioners, researchers, peer reviewers, and journal editors through international conferences and seminars. Secondly, as we have publicly registered this guideline on the EQUATOR Network, all relevant results and publications will be updated in real time. Thirdly, we will contact relevant journals for endorsement as journals play an important role in the implementation of reporting guidelines. Finally, we will monitor the application and evaluate the effect of PRISMA-M 2020 continuously and, when necessary, update it according to users’ feedback and the latest evidence.

Although guidelines do help improve the quality of reporting, there are some limitations to this extension. Firstly, although this checklist was completed through extensive solicitation of comments from CM clinicians, methodologists, journal editors, and epidemiologists, more than half of the experts were CM professionals, which may not be international enough for other traditional medicines. In future iterations of this guideline, we will optimize it by soliciting and incorporating comments from a broader group of experts from CAM societies. Secondly, in the development of PRISMA-M 2020, we did not include a large-scale user-based survey to test the practicality of each item. As the value of a guideline ultimately depends on its use, we will collect broad feedback from potential users (e.g., authors, editors, and reviewers of SRs) and update it accordingly. Despite these limitations, the PRISMA-M 2020 guideline has value as the first consensus-based reporting recommendations for SRs on moxibustion. We hope that these recommendations will promote better reporting and influence the methodology design of SRs on moxibustion.

## Conclusion

PRISMA-M 2020 was developed to help authors improve the reporting quality of SRs studying moxibustion interventions. The checklist can also be used to evaluate the current condition of reporting and to help journals identify moxibustion SRs of higher quality. Together, we believe this guideline will be a useful tool to promote the transparent reporting of SRs with moxibustion, thus to achieve its better use in clinical practice.

## Supplementary Information

**Additional file 1. S**_1_. Search strategy and flow chart of the literature review. S_2_. Available published examples of reporting moxibustion SRs.

## Data Availability

All results data of this study are provided in the manuscript and supplementary files. Data regarding the specific experts’ comments is available from the corresponding author on receiving a reasonable request.

## Abbreviations

CM: Chinese medicine
SRs: Systematic reviews
PRISMA: Preferred Reporting Items for Systematic Reviews and Meta-Analyses
PRISMA-M 2020: PRISMA Extension for Moxibustion intervention 2020
EQUATOR: Enhancing the QUAlity and Transparency Of health Research
RCTs: Randomized controlled trials
STRICTOM: STandards for Reporting Interventions in Clinical Trials Of Moxibustion
CONSORT: Consolidated Standards of Reporting Trials
RIGHT: A Reporting Tool for Practice Guidelines in Health Care
E&E: Explanation and elaboration
TIDieR: Template for Intervention Description and Replication
PICOS: Participants, interventions, comparators, outcomes, and study design
WHO: World Health Organization
ISO: International Organization for Standardization
CHM: Chinese herbal medicines
CAM: Complementary and alternative medicine
