# Supplementary material for: PRISMA extension for moxibustion 2020: recommendations, explanation, and elaboration
Source: Syst Rev. 2020 Oct 25;9:247. doi: 10.1186/s13643-020-01502-7 (PMC7586688; doi:10.1186/s13643-020-01502-7)
Supplement: Supplementary file 1 — Additional file 1. S1. Search strategy and flow chart of the literature review. S2. Available published examples of reporting moxibustion SRs. [file 13643_2020_1502_MOESM1_ESM.docx]

**Supplementary information**

**Additional file 1.**

**S_1_: Search strategy and flow chart of the literature review**

(1) Search strategy for Ovid (Embase, MEDLINE, and Cochrane Library):

1 (((Tian or medicinal) adj3 (vesiculation or moxibustion or mora)) or Tian Jiu or Tianjiu).mp.

2 (((Sanfu or dog days) adj3 (vesiculation or moxibustion or mora)) or Sanfu#moxibustion or Sanfu or Sanfu Jiu or San Fu Jiu or Sanfujiu).mp.

3 (((Fapao or blister) adj3 (vesiculation or moxibustion or mora)) or Fapao#moxibustion or Fapao or Fapao Jiu or Fa Pao Jiu or Fapaojiu).mp.

4 ((medicine or drug or Medicine#separated) adj3 (vesiculation or moxibustion or mora)).mp.

5 ((Sanjiu adj3 (vesiculation or moxibustion or mora)) or Sanjiu#moxibustion or Sanjiu or Sanjiu Jiu or San Jiu Jiu or Sanjiujiu).mp.

6 (((Sanfu or dog day*) adj3 (sticker* or patch* or paste*)) or Sanfu or Sanfu Jiu or San Fu Jiu or Sanfujiu).mp.

7(((Baijiezi or Bai Jie Zi or White mustard seed) adj3 (vesiculation or moxibustion or mora)) or Baijiezi#moxibustion or Baijiezi or Baijiezi Jiu or Bai Jie Zi Jiu or Baijiezijiu).mp.

8 (((Maogen or Japan Buttercup) adj3 (vesiculation or moxibustion or mora)) or Maogen#moxibustion or Maogen or Maogen Jiu or Mao Gen Jiu or Maogenjiu).mp.

9 (((Banmao or Chinese Blistering Beetle) adj3 (vesiculation or moxibustion or mora)) or Banbao#moxibustion or Banmao or Banmao Jiu or Ban Mao Jiu or Banmaojiu).mp.

10 (((Hanlian or Eclipta) adj3 (vesiculation or moxibustion or mora)) or Hanlian#moxibustion or Hanlian or Hanlian Jiu or Han Lian Jiu or Hanlianjiu).mp.

11 (((Suanni or Garlic) adj3 (vesiculation or moxibustion or mora)) or Suanni#moxibustion or Suanni or Suanni Jiu or Suan Ni Jiu or Suannijiu).mp.

12 (((Tiannanxing or Araceae or Arisaema*) adj3 (vesiculation or moxibustion or mora)) or Tiannanxing#moxibustion or Tiannanxing or Tiannanxing Jiu or Tian Nan Xing Jiu).mp.

13 ((Herbal or Acupoint or acupuncture) adj3 (Patch or Applicator or Application or sticking)).mp. [mp=title, abstract, original title, name of substance word, subject heading word, keyword heading word, protocol supplementary concept word, rare disease supplementary concept word, unique identifier] {Including Limited Related Terms}

14 or/1-13

15 limit 14 to systematic reviews [Limit not valid in CDSR; records were retained]

16 limit 14 to meta analysis [Limit not valid in CDSR; records were retained]

17 15 or 16

(2) Search strategy for CNKI:

SU=('系统综述'+'系统评价'+ 'Meta分析'+'Meta 分析'+'Meta-分析'+'荟萃分析'+'元分析'+'荟萃评价'+'合并分析'+'汇总分析'+'循证评价') and SU=('灸法'+'艾灸' +'天灸'+'药物灸'+'三伏灸'+'三九灸'+'三伏贴'+'白芥子灸'+'天南星灸' +'发泡灸'+'毛茛灸'+'斑蝥灸'+'蒜泥灸'+'针灸')

(3) Search strategy for VIP:

(M=系统综述+M=系统评价+ M=Meta分析+M=Meta 分析+M=Meta-分析+M=荟萃分析+M=元分析+M= 荟萃评价+M=合并分析+M= 汇总评价+M=循证评价)* (M=灸法+M=艾灸+M=天灸+ M=药物灸+M=三伏灸+M=三九灸+M=三伏贴+M=白芥子灸+M=天南星灸+M=发泡灸+ M=毛茛灸+M=斑蝥灸+M=蒜泥灸+M=针灸)

(4) Flow chart of data selection:


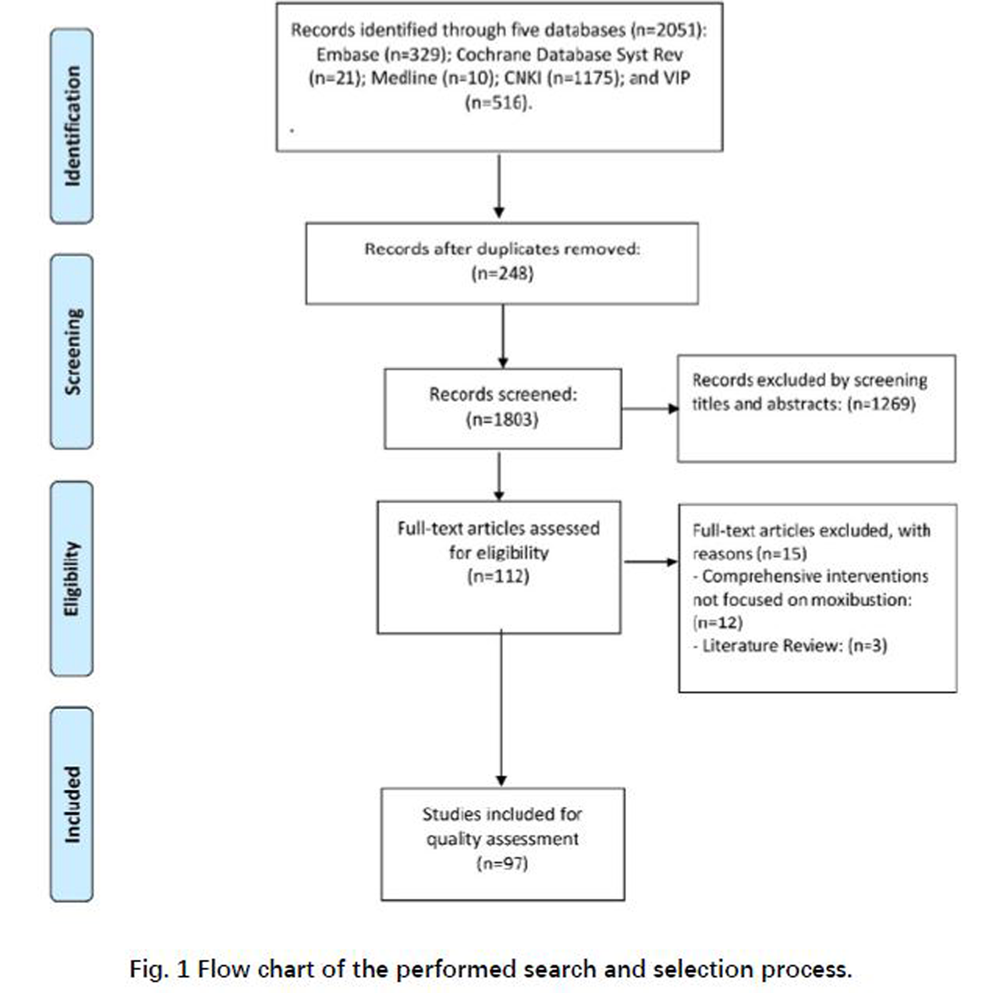


**S_2_: Available published examples of reporting moxibustion systematic reviews**

**Item 1. Title**

1. Identify the report as a systematic review, meta-analysis, or both.

Extensions: 1a. Statement of the specific type of moxibustion treatment, such as direct moxibustion or heat-sensitive moxibustion. 1b. Statement of whether the review targets the 1) Western medicine–defined disease(s), or 2) Western medicine–defined disease(s) with specific CM Pattern(s), or 3) CM Pattern(s), if applicable.

- *Example 1:* “Meta-Analysis on Heat Sensitive Moxibustion Therapy for chronic prostatitis” [1]
- *Example 2:* “Acupuncture and moxibustion for chronic fatigue syndrome in traditional Chinese medicine: a systematic review and meta-analysis” [2]
- *Example 3:* “Tian Jiu Therapy for the treatment of asthma in adult patients: A Meta-Analysis” [3]

*Note: No available examples for SR titles that included reporting of CM Pattern. For reference, we provide similar titles of clinical trials in the Explanation texts of this item.*

**Item 2. Abstract. Structured summary**

2. Provide a structured summary including, as applicable: background; objectives; data sources; study eligibility criteria, participants, and interventions; study appraisal and synthesis methods; results; limitations; conclusions and implications of key findings; systematic review registration number.

Extension: None.

- *Example 1:* “Objectives: To review and evaluate the efﬁcacy and safety of tian jiu therapy on san fu tian for adults with asthma. Methods: A literature search through August 31, 2013, was done to identify comparative studies evaluating effective rate, pulmonary function, immune response, recurrence rate, quality of life, and adverse events. The Cochrane Library, PubMed, EMBASE, and Chinese National Knowledge Infrastructure were searched; only randomized controlled trials with treatment groups using tian jiu therapy were included. Cochrane Collaboration’s risk of bias tool and Review Manager software, version 5.2, were used for the data synthesis. Results: Six studies involving 657 patients were identiﬁed. tian jiu therapy was more effective than the control intervention (odds ratio [OR], 3.51; 95% conﬁdence interval [CI], 2.05–6.00; p <0.00001; I2 =18%). The treatment group had a bigger decrease in IgE level (standard mean difference [SMD], -1.40; 95% CI, -2.18 to -0.63; p =0.0004; I2 =85%) and Eosinophil (Eos) level (SMD, -4.26; 95% CI, -6.28 to -2.23; p <0.00001; I2 =91%) compared with the control group. Included studies had a high risk of bias. Few adverse effects were reported in the included studies, and no serious adverse responses occurred. Adverse effects did not result in any dropouts. Conclusions: All studies indicated that tian jiu therapy has a positive effect on adults with asthma and that it is relatively safe because of its noninvasive nature. However, the limitations of the research design of the existing studies resulted in high risk of bias. More randomized controlled trials of better methodologic quality are needed to further conﬁrm efﬁcacy and safety of this therapy.” [3]

**Item 3. Rationale**

3. Describe the rationale for the review in the context of what is already known.

Extension: Describe the rationale for what is already known about moxibustion utilized for the target disease and/or CM Pattern (if any). If applicable, relevant theory of CM should be included.

- *Example 1: “*Moxibustion, as a treatment of acupuncture and moxibustion, refers to the ignition of moxa velvet or stick, then it acts on the corresponding acupoints for burning or fumigation, relying on the role of heat and medicine to achieve the role of disease prevention and treatment. Traditional Chinese medicine (TCM) theory holds that moxibustion has the functions of warming and dredging channels and collaterals, regulating qi and blood, dispelling cold and relieving pain, preventing and treating diseases, and strengthening health. It is often used in gynecologic uterine cold caused by infertility, menstrual pain, and metrorrhagia. Modern medical research suggests that moxibustion can improve ovarian function by inhibiting apoptotic events of naturally aging ovaries and enhancing antioxidant defense ability. The stimulation of meridian acupoints by its physical thermo-thermal characteristics and chemical composition of tar from Artemisia argyi leaves can activate the self-discipline movement of blood vessels, accelerate blood flow, improve blood circulation, and accelerate local blood circulation. Also, animal experiments have found that moxibustion can reduce the expression of p-PI3K, p-Akt, and p-mTOR in rat ovaries. It is suggested that moxibustion may improve ovarian hormone level and inflammatory response by inhibiting the PI3K/Akt/mTOR signaling pathway. Therefore, based on the above experiments, moxibustion has been widely applied to treat female infertility caused by polycystic ovary syndrome, premature ovarian failure, tubal obstruction, and so on.” [4]
- *Example 2:* “Moxibustion is a critical intervention in traditional Chinese medicine (TCM). The ancient classics that first record moxibustion are “Moxibustion Therapy on the Eleven Meridians of Yin and Yang” and “Moxibustion Therapy on the Eleven Meridians of Legs and Arms” which were written 2000 years ago. Moxibustion delivers heat stimulation at acupoints on the body surface by burning moxa leaves or moxa floss, with treatment effects in various diseases. There are 2 types of moxibustion in clinical practice: direct moxibustion, which directly applies heat simulation to the skin, and indirect moxibustion, which insulates heat simulation by various materials (e.g., ginger, garlic, or salt). Moxibustion is widely used in East Asia and is recommended for treating chronic inflammation and pain diseases, including osteoarthritis, lumbago, allergic asthma, dysmenorrhea, and chronic prostatitis/chronic pelvic pain syndrome (CP/CPPS), by the World Health Organization Advisory Committee on Acupuncture and Moxibustion. The analgesic and anti-inflammatory effects of moxibustion for CP/CPPS have been proved by several animal and human studies……” [5]

**Item 4. Objectives**

4. Provide an explicit statement of questions being addressed with reference to participants, interventions, comparisons, outcomes, and study design (PICOS).

Extension: None.

- *Example 1:* “This study attempts to evaluate the effect of acupuncture and moxibustion on chronic fatigue syndrome (CFS) systematically compared with other treatments by traditional and network meta-analysis.” [2]

**Item 5. Protocol and registration**

5. Indicate if a review protocol exists, if and where it can be accessed (e.g., Web address), and, if available, provide registration information including registration number.

Extension: None.

- *Example 1:* “Our SR has been registered on PROSPERO (registration number is CRD42019135593) and the protocol is designed strictly in coordinate with the preferred reporting items of the systematic review and meta-analysis protocol (PRISMA-P)…….” [4]

**Item 6. Eligibility criteria**

6. Specify study characteristics (e.g., PICOS, length of follow-up) and report characteristics (e.g., years considered, language, publication status) used as criteria for eligibility, giving rationale.

Extensions: 6a. Describe the diagnostic criteria of the target condition in Western medicine, and/or CM Pattern (if any). All criteria utilized should be universally recognized, or reference(s) where detailed explanation can be found should be given.

- *Example 1: “*Patients with physician-diagnosed allergic rhinitis (repetitive sneezing, watery rhinorrhoea, nasal congestion, an itchy nose and throat, and itchy and watery eyes) will be included in this review; there will be no sex, age, source or course restrictions.” [6]
- *Example 2: “*Studies will be included if they satisfy the following criteria: diagnosed with Primary dysmenorrhea (according to the Primary Dysmenorrhea Consensus Guidelines)……” [7]
- *Example 3:* “Participants with definite diagnostic criteria of constipation, regardless of age, sex, ethnicity, nationality, original of the case, course or cause of the disease. The diagnostic criteria of constipation should follow the International Rome III or Chinese medicine Criteria for the diagnosis and treatment of disease and syndrome. In addition, other criteria which equivalent to the above standards will also be included after the researcher’s specific assessment.” [8]

6b. Specify the types of moxibustion to be included, such as moxa burner moxibustion, natural moxibustion, or heat-sensitive moxibustion.

- *Example 1: “*Any type of moxibustion will be included, regardless of the treatment frequency, duration, material, type, and method. Studies involving direct moxibustion, indirect moxibustion, warm needling, moxa-burner moxibustion, heat sensitive moxibustion, natural moxibustion, herbal patching, and crude drug moxibustion will also be included. Research that compares different moxibustion materials, doses, or durations of moxibustion treatment will not be included. Studies that evaluated the effect of moxibustion with at least 1 day of follow-up will be included.” [9]
- *Example 2: “*Target interventions were restricted to pure moxibustion, such as moxa-stick moxibustion (MSM), moxa-cone moxibustion (MCM), box-appliance moxibustion (BAM) or heat sensitive moxibustion (HSM)……” [10]
- *Example 3: “*The treatment groups received tian jiu therapy, in which selected Chinese herbal medicines were pasted on selected acupoints on san fu tian (drugs and foundation treatment were allowed during acute asthma attack)……Studies that used combination therapies for the treatment group or were designed to compare the efficacy of tian jiu in different seasons were excluded.” [3]

6c. State whether CM-related outcome(s) were included, such as the change of degree and scope of symptoms and signs related to CM Pattern, or validated Pattern survey, if applicable.

- *Example 1: “*The outcomes were pain assessment (evaluation tool is visual analogue scale (VAS)), effective rate (the effectiveness was evaluated referring to “criteria of diagnosis and therapeutic effect of diseases and syndromes in traditional Chinese medicine,” which is made by the State Administration of Traditional Chinese Medicine in China. The effectiveness includes three grades: A cure: after treatment, lower abdominal pain and other accompanying symptoms disappeared and did not recur 3 menstrual cycles later after stopping treatment; B effectiveness: lower abdominal pain and other accompanying symptoms relieved, such as nausea, vomiting, diarrhea, cold sweat, and peripheral coldness; C treatment failure: lower abdominal pain and other accompanying symptoms were not alleviated. Moxibustion is a kind of traditional Chinese therapy, so the effective rate that referred to this standard is appropriate, the effective rate = cases (cure + effectiveness)/total cases, and the level of PGF2𝛼 in serum.” [7]
- *Example 2: “*The primary outcome measure was the clinical effective rate. It was a dichotomous outcome and the overall effectiveness of moxibustion therapy as a subjective assessment, which was defined as the proportion of participants who got improved in sleep quality and was based on response evaluation criteria used in the treatment of insomnia with traditional Chinese medicine (TCM). What’s more, it was reported by trial participants themselves. For example, clinical therapeutic effect criteria was categorized as cure, markedly effective, effective, or ineffective. according to the Guideline for Clinical Trials of New Patent Chinese medicines (GCTNPCM) evaluation standards, which define: (1) clinical cure as sleep time to restore normal sleep time OR a nighttime sleep duration of < 6 h, deep sleep, and full of energy after waking up; (2) markedly effective as significant improvement of insomnia, sleep time increased < 3 h compared to previous sleep time and an increase in the depth of sleep; (3) effective as amelioration in symptoms as sleep time increased < 3 h compared with the previous sleep time; and (4) ineffective as no significant improvement of insomnia OR deteriorated after treatment. Then the patients of “cure, markedly effective, effective” were taken as people who got improved in sleep quality and the patients of “ineffective” were taken as people who got unimproved in sleep quality. The total number of “cure, markedly effective, effective” were used to calculate effective rate. Other assessment criteria of clinical therapeutic effect with comparable definitions were also considered.” [11]

**Item 7. Information sources**

7. Describe all information sources (e.g., databases with dates of coverage, contact with study authors to identify additional studies) in the search and date last searched.

Extension: None.

- *Example 1:* “We will use computers to search PubMed, Medline, Embase, Web of Science and the Cochrane Central Register of Controlled Trials. Besides, China National Knowledge Infrastructure, China Biomedical Literature Database, China Science Journal Database, and Wan-fang Database will also be collected by our researchers. All databases will be searched from the date of creation to May 31, 2019……Besides, we will search a reference list to identify

published journals, books, conference articles, and gray literature related to the research topic.” [4]

**Item 8. Search**

8. Present full electronic search strategy for at least one database, including any limits used, such that it could be repeated.

Extension: None.

- *Example 1:* “The following search terms will be used: infertility, female infertility, polycystic ovary syndrome infertility, premature ovarian failure infertility, tubal infertility, ovulation barrier infertility; moxibustion, moxa leaf, moxa velvet, moxa stick, moxa cone, moxibustion box, ginger-separated moxibustion, dragon moxibustion (Du meridian moxibustion), cake-separated moxibustion, heat-sensitive moxibustion, medicinal moxibustion, sparrow pecking moxibustion, suspension moxibustion; in vitro fertilization, embryo transfer, IVF-ET. The sample search strategy in Table 1 will be used for PubMed. This search strategy will be slightly modiﬁed and used in several other databases.” [4]

**Item 9. Study selection**

9. State the process for selecting studies (i.e., screening, eligibility, included in systematic review, and, if applicable, included in the meta-analysis).

Extension: None.

- *Example 1:* “Two reviewers (LY and WL) independently complete the screening of documents and then cross-check to determine the ﬁnal inclusion of documents. In the ﬁrst stage, all documents after software review will be screened for title, summary, and keywords to determine which documents meet the selection criteria. In the second stage, we will evaluate the full text of the remaining studies and determine whether it meets the SRs criteria. The research excluded after reading the full text will also be documented, and the reasons for exclusion will be recorded. When differences arise in this process, we will invite third parties (ZQH) to arbitrate. The research ﬂow chart is shown in Fig. 1.” [4]

**Item 10. Data collection process**

10. Describe method of data extraction from reports (e.g., piloted forms, independently, in duplicate) and any processes for obtaining and confirming data from investigators.

Extension: None.

- *Example 1:* “We will produce an Excel spreadsheet to extract literature data, including the ﬁrst author, country, year of publication, patient characteristics, course of the disease, number of studies, interventions, course of the intervention, outcome indicators, main conclusions, conﬂicts of interest, recurrence rate, acupoint selection, and adverse events. If the data reported in the document is insufﬁcient, we will contact the author of the experiment for consultation and resolution. However, if we fail to contact the author, the document will be excluded.” [4]

**Item 11. Data items**

11. List and define all variables for which data were sought (e.g., PICOS, funding sources) and any assumptions and simplifications made.

Extensions: 11a. List and define the data of CM Pattern(s) in detail, considering the inclusion and exclusion criteria, if applicable.

- *Example 1:* “Two review authors (xx and xx) independently carried out data extraction, using a pre-tested data extraction form…… Participants: inclusion and exclusion criteria, total number enrolled and number in each comparison group, baseline characteristics, setting.” [12]
- *Example 2: “*The data from each included trial were extracted and recorded in a data extraction form by two authors (xx and xx) separately. The following factors were analyzed: …… participants (baseline characteristics, inclusion criteria, exclusion criteria, and sample size)……” [13]

11b. List and define the data of moxibustion interventions and controls (e.g. sham moxibustion), give details referring to STRICTOM and TIDieR.

- *Example 1: “*For the extraction of intervention-related information, the revised STRICTOM items were used to describe the details of moxibustion treatments used in the study context.” [14]
- *Example 2: “*To identify the therapeutic details of moxibustion, the following information was also extracted: type of moxibustion, acupuncture points used for moxibustion, treatment session and frequency……” [15]

11c. List and define the data of CM Pattern outcome(s), considering the methods and timepoints, if applicable.

- *Example 1: “*Two review authors (xx and xx) independently carried out data extraction, using a pre-tested data extraction form……Outcomes reported: the incidence and severity of chemotherapy-or radiotherapy-related toxicities, QoL, patient-reported physical and psychological indices of symptom distress based on a validated scale, any other objective outcome measures aimed at assessing side effects of chemotherapy or radiotherapy, modification or cessation of cancer treatments as the result of side effects or adverse effects, and incidence and types of adverse events resulting from moxibustion. For each outcome: outcome definition (with diagnostic criteria if relevant). Unit of measurement (if relevant).” [12]
- *Example 2: “*O (outcomes): The primary outcome included in this review was the efficacy of response of Rheumatoid arthritis (RA) to treatment with moxibustion by the American College of Rheumatology (ACR) outcome measures ACR20, 50 and 70…… In addition, the total response rate which is mostly based on the guiding principles of clinical research on new drugs of traditional Chinese medicine was also a primary outcome in this review. Similar to ACR rate, response rate includes a count of tender and swollen joints, morning stiffness duration, mean grip strength, patient assessment of global pain and laboratory parameters (erythrocyte sedimentation rate, C-reactive protein and rheumatoid factor). The secondary outcome included Disease Activity Score-28 (DAS28)……” [16]

**Item 12. Risk of bias in individual studies**

12. Describe methods used for assessing risk of bias of individual studies (including specification of whether this was done at the study or outcome level), and how this information is to be used in any data synthesis.

Extension: None.

- *Example 1:* “The Cochrane Manual V.5.1.0 tool will be used to assess the risk of bias for each included study. The evaluation includes random sequence generation, allocation sequence hiding, blind evaluation, incomplete result data, selective result report, and other bias sources. The assessment results will be divided into 3 levels: low risk, high risk, and uncertainty risk.’ [4]

**Item 13. Summary measures**

13. State the principal summary measures (e.g., risk ratio, difference in means).

Extension: None.

- *Example 1:* “For binary outcomes, the Mantel-Haenszel method for odds ratio (OR) with corresponding 95% conﬁdence intervals (CIs) was used. For continuous data, standard mean difference (SMD) with corresponding 95% CI was used……” [3]

**Item 14. Synthesis of results**

14. Describe the methods of handling data and combining results of studies, if done, including measures of consistency (e.g., I2) for each meta-analysis.

Extension: None.

- *Example 1:* “Heterogeneity among studies was measured by using I2 statistics; when the heterogeneity test showed an I2 of £50%, a ﬁxed-effects model was used, and when the heterogeneity test showed an I2 of 50%, a random-effects model was used.” [3]

**Item 15. Risk of bias across studies**

15. Specify any assessment of risk of bias that may affect the cumulative evidence (e.g., publication bias, selective reporting within studies).

Extension: None.

- *Example 1:* “We will conduct a sensitivity analysis to test the recklessness of major decisions in the review process. The main contents of the analysis include the impact of method quality, sample size, and missing data on the study. The meta-analysis will be reused and poor quality research will be excluded. The results will be compared and discussed according to the results.” [4]

**Item 16. Additional analyses**

16. Describe methods of additional analyses (e.g., sensitivity or subgroup analyses, meta-regression), if done, indicating which were pre-specified.

Extension: Describe methods of subgroup analyses in terms of different types of included moxibustion interventions and/or included CM Pattern participants (if applicable), if done, indicating which were pre-specified.

- *Example 1: “*In the case of high heterogeneity, we will determine the source of heterogeneity by subgroup analysis according to the different combined intervention methods, different treatment courses, different amount of moxibustion, different single moxibustion treatment time, and other different influence factors of female infertility.” [4]
- *Example 2: “*We will investigate whether heterogeneity is caused by the following confounders through subgroup analysis: (1) Type of moxibustion: direct moxibustion versus indirect moxibustion. (2) Course of disease: <1 year versus ≥1 year. (3) Length of follow up: <3 months versus ≥3 months.” [5]
- *Example 3: “*If the necessary data are available, subgroup analyses will be carried out according to different factors as follows: 1. Control interventions (eg, sham/placebo moxibustion, no treatment, other TCM treatment or non-TCM treatment); 2. Type of moxibustion (eg, direct moxibustion, indirect moxibustion, heat-sensitive moxibustion, moxa burner moxibustion, warm needling moxibustion, crude drug moxibustion or natural moxibustion); 3. Treatment frequency (eg, less than three times per week versus more than three times per week); 4. Classification of AR (eg, intermittent, persistent, seasonal or PAR).” [6]
- *Example 4: “*To identify heterogeneity between the included studies, a subgroup analysis will be conducted if there is a sufficient number of articles in each subgroup. The criteria of a subgroup analysis will be as follows: (1) disease duration, such as chronic (>3 months) or acute lower back pain (we will conduct a subgroup analysis according to disease duration even though there are not sufficient number of included studies); (2) type of control group, such as placebo moxibustion, conventional treatment, other TCM treatment, and no treatment; (3) type of moxibustion, such as direct moxibustion, indirect moxibustion, warm needling moxibustion, moxa burner moxibustion, heat sensitive moxibustion, and crude drug moxibustion; (4) species of herb used in the moxibustion treatment; and (5) treatment number, frequency, and duration.” [9]

**Item 17. Study selection**

17. Give numbers of studies screened, assessed for eligibility, and included in the review, with reasons for exclusions at each stage, ideally with a flow diagram.

Extension: None.

- *Example 1:* “A total of 1345 potentially relevant citations were identified, 47 of which were degree research thesis. 157 duplicate papers were removed firstly, and 1000 papers were excluded after scanning their titles and abstracts. After screening the full texts of the included articles, 158 studies were excluded for the following reasons: no relevant data (n = 35), inapposite treatments set (n = 73), patients with tuberculosis (n = 1), duplicate reports (n = 12), not RCT (n = 3), unclear diagnose criteria (n = 7), inapposite criteria of invalid evaluation (n = 24), inappropriate western medicine (n = 3). However, one additional article was identified during screening. Finally, 31 studies (including1 three-arm study) were included for further analysis (Fig. 1).” [2]

**Item 18. Study characteristics**

18. For each study, present characteristics for which data were extracted (e.g., study size, PICOS, follow-up period) and provide the citations.

Extensions: 18a. Present characteristics for the data of participants which include CM Pattern(s), considering 1) diagnostic criteria; 2) baseline data, if applicable.

- *Example 1: “*The general characteristics of included trials are presented in Table 1……The degree of disease was mainly moderate to severe. The duration of primary dysmenorrhea (PD) ranged from half a year to 15 years. The main symptoms were cold and damp accumulation, qi stagnation, and blood stasis……” [7]
- *Example 2: “*The diagnostic criteria used in the 22 trials included (1) Chinese classification and criteria for mental disorders 3rd edition (CCMD-3), (2) Chinese classification and diagnostic criteria for mental disorders second edition-revision (CCMD-2-R), (3) GCTNPCM, (4) International Classification of Disease 10th Version (ICD-10), (5) sleep efficiency calculation of the World Health Organization (WHO), (6) Criteria of Diagnosis and Therapeutic Effects for TCM Disease and Syndrome (CDT&ETCMD&S), (7) Pittsburgh Sleep Quality Index (PSQI), and (8) unclear criteria.” [11]
- *Example 3: “*Participants in studies were diagnosed with osteoarthritis (OA) according to American College Rheumatology (ACR) criteria in seven trials, Chinese Medical Association (CMA) criteria in four trials and the Guiding Principles of Clinical Research on New Drugs for Traditional Chinese Medicine (GP-TCM) in seven trials……The detailsof the treatment regimens are summarized in Table 3.” [14]

18b. Present characteristics for the data of moxibustion intervention(s) and controls (e.g. sham moxibustion) for each study referring to STRICTOM and TIDieR.

- *Example 1: “*Moxibustion interventions: All of the RCTs had used a combination of indirect moxibustion and routine care. Two RCTs used moxa sticks, one applied ginger cake-separated moxas, and the remaining study used both moxibustion methods. In all of the RCTs, the selection of the acupuncture point for moxibustion treatment had been based on the traditional Chinese medicine (TCM) theory, and the rationale behind the selection was described in each article. The time of each moxibustion treatment ranged from 5 to 30 min, and the total number of treatment sessions varied from 14 to 40. Two RCTs used fixed acupuncture points, and the others used both fixed and individual points determined by the practitioner. The details of moxibustion regimens are summarized in Table 2…… Three items (style of moxibustion, reasoning for choosing treatment details, and treatment regimen respectively corresponding with STRICTA 1a, 1b, and 3) were well-stated in all articles. Only two RCTs described the feature of moxibustion, procedure and technique of moxibustion treatment, and control treatment in detail. The background of the practitioner and information to patients were not described in any of the articles. These findings are presented in Online Resource 3.” [17]
- *Example 2:* “Warm-needle moxibustion therapy protocols of included trials: All studies clearly described protocols in warm-needle moxibustion therapy, such as location of operation, selected acupoints for intervention, intervention methods, degree of operation, and treatment duration. All studies reported that warm-needle moxibustion was performed on points that lie in the limbs, or the limbs and the governing meridian. The acupoints are distributed mainly in the Large Intestine Channel of Hand Yang Ming (LI) and the Spleen Meridian of Foot Tai Yin (SP), such as Quchi LI 11, Jianyu LI 15, and Xuehai SP 10. In eight studies, all selected points received warm-needle moxibustion. In four studies, the main points were given warm-needle moxibustion, whereas adjunct points were given acupuncture. Nine studies used 5–11 acupoints for warm-needle moxibustion to study effects on upper and lower limbs. Two studies used three to five acupoints to observe effects of warm-needle moxibustion on upper limbs, and only Liu used four acupoints to observe effects on lower limbs. All studies described the dosage, frequency, and duration of warm-needle moxibustion. All studies adopted one to three moxa cones or sticks for each acupoint, and patients perceived needling sensations for 20–30 min per intervention. Frequency of treatment was once a day in all studies, but total treatment duration was not equal and varied from 12 to 60 days (Table 2).” [18]
- *Example 3:* “Moxibustion included heat-sensitive moxibustion (5 trials), mild moxibustion (4 trials), du-moxibustion (4 trials) based on the specific acupoints. The frequency of moxibustion covered in the included trials was varied, but the majority of studies chose to moxibustion treatment once daily. Specific acupoints of the moxibustion were shown in Table 2……The control groups only used conventional treatments, including alendronate sodium, calcium supplementation, salmon calcitonin, calcium supplementation, alendronate sodium, α-D3, combined with resistance training, calcium supplementation and alendronate sodium, calcium supplementation, alendronate sodium and calcitriol. However, the type of study design such as moxibustion vs. no treatment or waiting-list was not found. The treatment duration ranged from 14 days to 12 months, but most of studies (62%, 8/13) designed a 3-month treatment program in clinical trials.” [19]
- *Example 4:* “In the control groups of the 22 included trials, the treatment methods included 2 western medications (Estazolam, Diazepam plus Oryzanol plus VB1), 6 oral Chinese medicine therapies (Sanhuang Anshen decoction, Renshenguipi pill, Huatanjieyu decoction, Tianwangbuxin decoction, Anshen Bunao decoction, Anshen Bunao Ye), and 5 other TCM therapies (head-acupoint massage, point-application, head-needle acupuncture, auricular-plaster therapy, and acupuncture).” [11]

18c. Present characteristics for the data of outcomes which include CM Pattern(s), considering 1) name and measuring methods; 2) measuring timepoints and length of follow-up, if applicable.

- *Example 1: “*Study characteristics: ……Five RCTs met the American College of Rheumatology (ACR) efficacy evaluation criteria, whereas the remaining trials only described Chinese medicine efficacy evaluation criteria. The details of the treatment regimens are summarized in Table 2.” [16]
- *Example 2: “*The effectiveness of moxibustion was classified according to 5 criteria: (1) Guideline for Clinical Trials of New Patent Chinese medicines (GCTNPCM), (2) Criteria of Diagnosis and Therapeutic Effects for TCM Disease and Syndrome (CDT&ETCMD&S), (3) Pittsburgh Sleep Quality Index (PSQI), (4) WHO sleep efficiency calculation, (5) unclear criteria.” [11]

**Item 19. Risk of bias within studies**

19. Present data on risk of bias of each study and, if available, any outcome-level assessment (see Item 12).

Extension: None.

- *Example 1:* “After assessing the quality of studies based on the basis of Cochrane risk of bias, we revealed that 17 trials reported random sequence generation, 2 studies provided information on allocation concealment, 4 trials described blinding of participants (single or double),1 trial described blinding of outcome assessment,3 trials had unclear bias of complete data and 5 trials had unclear other biases (defined as baseline data comparability). Selective reporting bias was unclear (Fig. 2).” [2]

**Item 20. Results of individual studies**

20. For all outcomes considered (benefits or harms), present, for each study: (a) simple summary data for each intervention group and (b) effect estimates and confidence intervals, ideally with a forest plot.

Extension: None.

- *Example 1:* “Effective Rate. The effective rate of clinical treatment was pooled for 7 studies. Heterogeneity among studies was low (𝑃 = 0.14, 𝐼2 = 38%); thus, a fixed-effect model was employed. There was significant difference between moxibustion and non-moxibustion treatments on increasing the total effective rate (RR = 1.16, 95% CI (1.06, 1.27), 𝑃 = 0.001) (Figure 2). ” [7]

**Item 21. Synthesis of results**

21. Present results of each meta-analysis done, including confidence intervals and measures of consistency.

Extension: None.

- *Example 1:* “Synthesis of results: CbAM versus Chinese herbal medicine: ……The pooled results of the 8 trials that compared CbAM with Chinese herbal medicine were of RR (95% CI), 1.17 (1.09-1.25)……(Fig. 3). There were no heterogeneity (I^2^ = 0%, p = 0.83)……SAM versus Chinese herbal medicine……” [2]

**Item 22. Risk of bias across studies**

22. Present results of any assessment of risk of bias across studies (see Item 15).

Extension: None.

- *Example 1:* “Seven studies’ publication bias was assessed for the outcome of effective rate by using Stata 12.0 software. Begg’s test results showed there was no publication bias [𝑧 = 1.20 (continuity corrected) Pr > |𝑧| = 0.230 (continuity corrected), 0.230 > 0.05]. The other studies’ publication bias was judged as unclear because of the limited number of trials.” [7]

**Item 23. Additional analysis**

23. Give results of additional analyses, if done (e.g., sensitivity or subgroup analyses, meta-regression [see Item 16]).

Extension: Give results of subgroup analyses based on the different types of moxibustion interventions and participants with CM Patterns (if any), if done.

- *Example 1:* “The subgroup meta-analysis (Fig. 3) demonstrated that moxibustion was more effective for insomnia than western medications (RR = 1.16, 95 % CI 1.09 to 1.24, P < 0.00001), oral Chinese medicine (RR = 1.11, 95 % CI 1.04 to 1.18, P = 0.002), and other TCM therapies (RR = 1.22, 95 % CI 1.15 to 1.30, P < 0.00001)……To account for clinical heterogeneity and subgroup differences probably arising from the use of different criteria to evaluate the effectiveness of moxibustion therapy for insomnia, a sensitivity analysis of trials using only Guideline for Clinical Trials of New Patent Chinese medicines (GCTNPCM) criteria was conducted. The effectiveness of moxibustion classified according to GCTNPCM criteria was described in 12 trials (moxibustion vs. western medications in 3 trials, moxibustion vs. oral Chinese medicine in 3 trials and moxibustion vs. other TCM therapies in 6 trials)…..” [11]
- *Example 2: “*Subgroup Analysis: In order to analyze if there was difference on clinical curative effect of moxa moxibustion at different intervention times to treat primary dysmenorrhea (PD), subgroup analysis was performed for the outcome of effective rate. The subgroup meta-analysis showed there was no statistical significance among 3 days, 5 days, 7 days, or two weeks before menstruation to start the moxa moxibustion therapy on improving the effective rate (𝑃 = 0.12) (Figure 5).” [7]

**Item 24. Summary of evidence**

24. Summarize the main findings including the strength of evidence for each main outcome; consider their relevance to key groups (e.g., health care providers, users, and policy makers).

Extension: None.

- *Example 1:* “In our meta-analysis, we included 31 trials for traditional and network meta-analysis. In traditional meta-analysis, CbAM and SAM were more effective than Chinese herbal medicine, western medicine and sham-acupuncture. In network meta-analysis, similar results were found. We also analysed all treatments by network meta-analysis, of which CbAM was the most effective, followed by SAM, Chinese herbal medicine, western medicine, and then sham-acupuncture……” [7]

**Item 25. Limitations**

25. Discuss limitations at study and outcome level (e.g., risk of bias), and at review level (e.g., incomplete retrieval of identified research, reporting bias).

Extension: None.

- *Example 1:* “First, diagnostic criteria for insomnia varied between included trials. As the objective of this study was to investigate the effectiveness of moxibustion for insomnia, subgroup analyses according to diagnostic criteria were not performed…….Second, overall risk of bias in the 22 included trials was high. Third, the included trials relied on different criteria to classify the effectiveness of moxibustion which could lead to heterogeneity between the trials or subgroup differences……However, a sensitivity analysis focusing on GCTNPCM criterion indicated the results of the meta-analysis were robust. Fourth, there was an obvious publication bias……” [11]

**Item 26. Conclusions**

26. Provide a general interpretation of the results in the context of other evidence, and implications for future research.

Extension: None.

- *Example 1:* “It is difficult to get the conclusion regarding the effectiveness and safety of moxibustion for primary insomnia due to insufficient evidence, such as the high risk of bias in the included studies, small sample sizes, and few reports on adverse effects. Moxibustion should be considered as a novel therapeutic option for insomnia, and more rigorous clinical trials of moxibustion therapy for insomnia are needed to assess its effects.” [11]

**Item 27. Funding**

27. Describe sources of funding for the systematic review and other support (e.g., supply of data); role of funders for the systematic review.

Extension: None.

- *Example 1:* “This research was supported by a grant of the Korea Health Technology R&D Project through the Korea Health Industry Development Institute (KHIDI), funded by the Ministry of Health & Welfare, Republic of Korea (HB16C0001). The funder had no role in study design, in the collection, analysis, interpretation of data, and decision to submit the manuscript for publication.” [15]

**References**

[1] Chen SD, Liu BP, Qian LH, Li J, Li SQ, He Y. Meta Analysis on Heat Sensitive Moxibustion Therapy for Chronic Prostatitis. *J Clin Acupunct Med*. 2015;31(12): 54-58.

[2] Wang T, Xu C, Pan K, Xiong H. Acupuncture and moxibustion for chronic fatigue syndrome in traditional Chinese medicine: a systematic review and meta-analysis. *BMC Complement Altern Med*. 2017;17(1):163. DOI: 10.1186/s12906-017-1647-x.

[3] Chan CW, Lee SC, Lo KC, Wong HK, Li L. Tian jiu therapy for the treatment of asthma in adult patients: a meta-analysis. *J Altern Complement Med*. 2015;21(4):200-207. DOI: 10.1089/acm.2014.0009.

[4] Hou T, Zheng Q, Feng X, Liu Y, Wang L, Li Y. Efficacy and safety of moxibustion in female infertility patients undergoing in vitro fertilization and embryo transfer: A systematic review protocol. *Medicine (Baltimore)*. 2019;98(44): e17560. DOI: 10.1097/MD.0000000000017560.

[5] Cao Q, Zhou X, Chen J, Zhong Y, Zhang H, Ao Q, et al. Efficacy and safety of moxibustion in patients with chronic prostatitis/chronic pelvic pain syndrome: A systematic review protocol. *Medicine (Baltimore)*. 2019;98(20): e15678. DOI: 10.1097/MD.0000000000015678.

[6] Chen S, Guo S, Wang J, Ha E, Marmori F, Wang Y, et al. Effectiveness of moxibustion for allergic rhinitis: protocol for a systematic review. *BMJ Open*. 2015;5(5): e006570. DOI: 10.1136/bmjopen-2014-006570.

[7] Gou CQ, Gao J, Wu CX, Bai DX, Mou HY, Hou XL, et al. Moxibustion for Primary Dysmenorrhea at Different Interventional Times: A Systematic Review and Meta-Analysis. *Evid Based Complement Alternat Med*. 2016;2016: 6706901. DOI: 10.1155/2016/6706901.

[8] Chen Q, Wang F, He WX, Yang SZ, Gao ZH, Wang LQ. A Meta-analysis of clinical efficacy of moxibustion on constipation. *J Lanzhou University (Medical Sciences)*. 2019;45(2): 17-25. [Article in Chinese]

[9] Leem J, Lee S, Park Y, Seo BK, Cho Y, Kang JW, et al. Effectiveness and safety of moxibustion treatment for non-specific lower back pain: protocol for a systematic review. *BMJ Open*. 2017;7(6): e014936. DOI: 10.1136/bmjopen-2016-014936.

[10] Zhong DK, Tang D, Xue L, Wen J, Li YP. Effectiveness of moxibustion for exercise-induced fatigue--a systematic review for randomized controlled trials. *Chin J Integr Med*. 2016;22(2): 130-140. DOI: 10.1007/s11655-014-1849-8.

[11] Sun YJ, Yuan JM, Yang ZM. Effectiveness and safety of moxibustion for primary insomnia: a systematic review and meta-analysis. *BMC Complement Altern Med*. 2016;16: 217. DOI: 10.1186/s12906-016-1179-9.

[12] Zhang HW, Lin ZX, Cheung F, Cho WC, Tang JL. Moxibustion for alleviating side effects of chemotherapy or radiotherapy in people with cancer. *Cochrane Database Syst Rev.* 2018;11:CD010559.

DOI: 10.1002/14651858.CD010559.pub2.

[13] Huang Z, Qin Z, Yao Q, Wang Y, Liu Z. Moxibustion for Chemotherapy-Induced Nausea and Vomiting: A Systematic Review and Meta-Analysis. *Evid Based Complement Alternat Med*. 2017; 2017: 9854893. DOI: 10.1155/2017/9854893.

[14] Choi TY, Lee MS, Kim JI, Zaslawski C. Moxibustion for the treatment of osteoarthritis: An updated systematic review and meta-analysis. *Maturitas*. 2017;100: 33-48. DOI: 10.1016/j.maturitas.2017.03.314.

[15] Han CH, Ma JN, An N, Yoon SG, Kim M. Moxibustion for stroke: Systematic review, meta-analysis, and GRADE-based recommendations. *Euro J Integr Med*. 2018;20: 115-125. DOI: <https://doi.org/10.1016/j.eujim.2018.04.008>

[16] Sun ZL, Xu X, Du SZ, Jiang X. Moxibustion for treating rheumatoid arthritis: A systematic review and meta-analysis of randomized controlled trials. *Euro J Integr Med*. 2014;6(6): 621-630.

DOI: <https://doi.org/10.1016/j.eujim.2014.08.002>

[17] Lee S, Jerng UM, Liu Y, Kang JW, Nam D, Lee JD. The effectiveness and safety of moxibustion for treating cancer-related fatigue: a systematic review and meta-analyses. *Support Care Cancer*. 2014;22(5):1429-1440. DOI: 10.1007/s00520-014-2161-z.

[18] Yang L, Tan JY, Ma H, Zhao H, Lai J, Chen JX, et al. Warm-needle moxibustion for spasticity after stroke: A systematic review of randomized controlled trials. *Int J Nurs Stud*. 2018;82: 129-138. DOI: 10.1016/j.ijnurstu.2018.03.013.

[19] Xu F, Huang M, Jin Y, Kong Q, Lei Z, Wei X. Moxibustion treatment for primary osteoporosis: A systematic review of randomized controlled trials. *PLoS One*. 2017;12(6): e0178688. DOI: 10.1371/journal.pone.0178688.
